# Controllably Alloyed, Low Density, Free-standing Ni-Co and Ni-Graphene Sponges for Electrocatalytic Water Splitting

**DOI:** 10.1038/srep31202

**Published:** 2016-08-11

**Authors:** Thazhe Veettil Vineesh, Suhail Mubarak, Myung Gwan Hahm, V. Prabu, Subbiah Alwarappan, Tharangattu N. Narayanan

**Affiliations:** 1 CSIR- Central Electrochemical Research Institute (CSIR-CECRI), Karaikudi – 630006, India; 2 TIFR- Centre for Interdisciplinary Sciences (TCIS), Tata Institute of Fundamental Research, Hyderabad – 500075, India; 3 Academy of Scientific & Innovative Research, Chennai - 600113, India; 4 Department of Materials Science and Engineering Inha University, 100 Inharo, Incheon, Nam-gu, 22212, Korea

## Abstract

Synthesis of low cost, durable and efficient electrocatalysts that support oxygen evolution reaction (OER) and hydrogen evolution reaction (HER) are the bottlenecks in water electrolysis. Here we propose a strategy for the development of controllably alloyed, porous, and low density nickel (Ni) and cobalt (Co) based alloys - whose electrocatalytic properties can be tuned to make them multifunctional. Ni and Co based alloy with the chemical structure of Ni_1_Co_2_ is identified as an efficient OER catalyst among other stoichiometric structures in terms of over potential @ 10 mAcm^−2^ (1.629 V), stability, low tafel slope (87.3 mV/dec), and high Faradaic efficiency (92%), and its OER performance is also found to be on par with the benchmarked IrO_2_. Tunability in the porous metal synthesis strategy allowed the incorporation of graphene during the Ni sponge formation, and the Ni- incorporated nitrogen doped graphene sponge (Ni-NG) is found to have very high HER activity. A water electrolysis cell fabricated and demonstrated with these freestanding electrodes is found to have high stability (>10 hours) and large current density (10 mAcm^−2^ @ 1.6 V), opening new avenues in the design and development of cost effective and light weight energy devices.

Rational organization and design of bulk porous multi-metallic architectures are highly demanding and receiving tremendous attention from the scientific community due to their immense applications in energetics^1^. On the other hand, advancement in atomically thin layered materials (2D materials), witnessed during the last decade, has initiated everlasting attempts towards the development of composites of metals/metal oxides and 2D materials based electrocatalysts^2^,^3^,^4^,^5^,^6^. Despite the development of numerous metal free 2D materials based catalysts, benchmarked conventional metals in energy technologies are still remain as unbeatable in terms of over potential and long-standing performance^7^,^8^,^9^. Nevertheless the expensive and scarce metals in conventional energetics need to be replaced/ reduced for viable futuristic energy technologies.

One of the ways for the efficient reduction of metal usage (‘metal management’) is the creation of porous metallic structures such as sponges or foams (with their 75–95% of the volume consists of void spaces) which will eventually help to decrease the weight of the device. Further, low density porous materials provide enhanced surface area than their bulk high density counter parts that can lead to large electrode-electrolyte contact areas and high specific and mass activities^10^,^11^,^12^. Various approaches were reported for the development of metallic foams (foams are hard and rigid, unlike sponges), where de-alloying, combustion synthesis, liquid polyol reduction method, decomposition of metal complexes etc. are to name a few^13^,^14^,^15^,^16^,^17^,^18^,^19^. All the above mentioned techniques rely on laborious multistep processes including casting in inert atmospheres. Here we propose a single step alloying process for the development of multi-metallic sponges using a template free bulk synthesis method, and demonstrated the performance of these sponges in water electrolysis.

Splitting of water into oxygen and hydrogen is identified as one of the promising alternatives to address the energy crisis *via* the production of electric energy and hydrogen fuel^19^,^20^,^21^. Efficient oxygen evolution reaction (OER) and hydrogen evolution reaction (HER) are the two key components in water splitting technology. OER and HER reaction kinetics are inherently sluggish and impose large over potentials. This results in to large losses in the overall efficiency of the respective devices, and hence catalysts need to be supplemented to improve the reaction kinetics. Though enormous efforts have been devoted to find effective catalysts, precious metals based catalysts such as RuO_2_ and IrO_2_ are still remain as unbeatable for OER^23^,^24^,^25^. However, these precious metal oxides are not suitable for large-scale applications and sustainable energy technologies. Development of active (low over potential), durable (stability in the performance), and inexpensive catalysts is therefore of high topical concern.

Among various abundant transition metals, Ni is probably the most studied catalytic material in its various forms^26^,^27^,^28^. Ni is a good electrode material in alkaline medium for OER due to its relatively low over potential, low cost and high corrosion resistance^29^,^30^,^31^. Further, spinel oxides of Co are also identified as good catalysts for OER^32^,^33^,^34^,^35^. Co (metallic and its other forms such as oxides or hydroxides) or Fe is often used as an additive to improve the reversibility, electrocatalytic activity and stability of pure Ni^36^,^37^,^38^,^39^,^40^,^41^. In the case of HER, the most successful catalysts identified to the date are mainly based on platinum based materials. Recently, the concept of engineering the same material by doping/alloying to make them enabled for bi- or tri- functional catalysis is gaining interest. For example, a recent report cite the possibility of metal/metal oxide/doped graphene composites for multifunctional catalysis - oxygen reduction, oxygen evolution and hydrogen evolution reactions, whose properties can be on par with commercial noble metal based catalysts^42^. Hence incorporation of porous graphene in to metallic sponges, if possible, can bring multiple functionalities in to graphene.

Herein, we report a one pot, economically feasible, and scalable synthesis method to produce Ni/Co based catalysts for various energy technologies including water splitting with ‘enhanced activity and appealing stability’ using readily available nickel and cobalt salts (a schematic of the concept is portrayed in Fig. 1). Different weight ratios of nickel nitrate hexahydrate [Ni (NO_3_)_2_.6H_2_O] and cobalt nitrate hexahydrate [Co (NO_3_)_2_.6H_2_O], are used as Ni and Co sources respectively, and ethylene glycol (EG) is used as both solvent and reducing agent. In a simple procedure, a sudden introduction of reactant solution [EG+ Ni (NO_3_)_2_+ Co (NO_3_)_2_)] in to a hot glass dish (maintained at a temperature of 300 °C) leads to the evolution of NO_2_ from the metal precursors and subsequent production of fluffy sponges. The HER catalyst is also developed using the same procedure with the introduction of nitrogen incorporated graphene (NG)during the reaction and it leads to the formation of highly stable nitrogen doped graphene wrapped nickel sponges (Ni-NG). Finally, a water electrolysis cell is demonstrated by using Ni-Co alloy as anode and Ni-NG as cathode.

## Results and Discussions

### Material synthesis and characterization

In the optimized synthesis procedure for Ni sponges, 36 g of Ni (NO_3_)_2_.6H_2_O (Sigma-Aldrich) and 18 mL of EG (C_2_H_6_O_2_) (Sigma-Aldrich) were taken in a 100 mL beaker and ultra-sonicated for 1 hour. The resultant mixture was poured into a cylindrical crystalline glass dish, which was pre-heated to a temperature of 300 °C using a hot plate in the ambient atmosphere. A sudden introduction of reactants in to the hot glass dish leads to the evolution of NO2 gas and subsequent production of fluffy Ni sponge. Here EG is found to be acting as reducing agent for the reduction of nickel (II) ion to metallic nickel^43^. The same synthesis route was followed for the preparation of Ni-Co alloy sponges. Here Ni (NO_3_)_2_.6H_2_O and Co(NO_3_)_2_.6H_2_O, were mixed together taking a total amount of 36 g in the weight ratio of 1:1, 1:2 and 2:1 respectively and the resulting sponges were named as Ni_1_Co_1_, Ni_1_Co_2_ and Ni_2_Co_1_(deduced from the crystal structure studied later using XRD, X-ray fluorescence (XRF), X-ray photoelectron spectroscopy (XPS), and energy dispersive X-ray spectroscopy (EDS)) respectively. Cobalt oxide (Co_3_O_4_) sponge was resulted from the reaction of Co(NO_3_)_2_.6H_2_O and EG in the absence of Ni salt, since the energy needed for the reduction of Co (II) to Co (0) is higher than that of Ni (II) to Ni (0)^44^.

Powder X-ray diffraction (Bruker XRD, CuKα radiation, λ= 1.5418 Å) experiments were performed on sponges before and after the electrochemical experiments as shown in Fig. 2A,B. The XRD pattern of the Ni sponge revealed that the peaks at 2θ values 44.8, 52.2 and 76.6 belong to (111), (200) and (220) planes respectively (JCPDS no: 870712) indicating the formation of face centered cubic (fcc) Ni. Ni sponge XRD shows the absence of oxide peaks in it (either nil or less than 2% of the detectable level of XRD). The XRD pattern of the Co_3_O_4_ sponge shows peaks at 19.09, 31.34, 36.89, 38.67, 44.2, 59.4, 65.37, 73.5 and 77.5 correspond to (111), (220), (222), (311), (400), (511), (440), (620) and (533) planes of Co_3_O_4_ (JCPDS card no. 42–1467). The XRD pattern of the all samples of Ni-Co sponges are showing peaks at angles 2θ = 44.48, 51.92 and 76.35 belong to planes (111), (200) and (220) respectively, which correspond to that of Ni-Co alloys^45^.

To find out the elemental composition of different Ni-Co alloys, XRF studies are conducted on sponges (Figure S1). The elemental composition (Ni:Co) in Ni_1_Co_1_, Ni_2_Co_1_ and Ni_1_Co_2_ are found to be 49.30:50.70, 66.36:33.64 and 32.99:67.01 respectively. To confirm further the observed elemental composition, EDS analysis is conducted on various sponges (Figure S2). The elemental compositions calculated from EDS are found to be in close agreement with the XRF values (49.50:50.50, 66.89:33.11 and 33.72:66.80 for Ni_1_Co_1_, Ni_2_Co_1_ and Ni_1_Co_2_ respectively).

The morphology of the as prepared binary Ni-Co alloy structures are examined via field emission scanning electron microscope (FESEM). Figure 2C–E shows the FESEM images of various sponges. It is clear from the figures that all the images exhibit the characteristics of interconnected porous network structure with a wide range of pore sizes (micro to mesoscopic pores). BET (supporting information, Figure S3A) surface area of the Ni_1_Co_2_ is calculated as ~10 m^2^/g which is much higher than that of commercial Ni sponge (0.01–0.1 m^2^/g). The isotherm shows a type-II adsorption isotherm (negligible concave section) - which is attributed to the microporous volume uptake while rapid rise in total volume near P/P_o_ = 1 corresponds to a microporous material (which further confirmed from Hg intrusion porosimetry, supporting information Figure S3B). The Hg porosimetry results show that the pore diameter varies from ~4 μm to 262 μm. FESEM images at different magnifications are shown in supporting information (Figure S4).

Though the morphologies of different sponges are same, there is a large variation in their calculated bulk densities (tapped density). Ni sponge is having lowest density and with the increase in Co to Ni ratio, the densities of the sponges are found to be increased. The densities of the sponges are found to be 45 mg/cc, 60 mg/cc, 72 mg/cc, 100 mg/cc and 122 mg/cc for Ni, Ni_2_Co_1_, Ni_1_Co_1_, Ni_1_Co_2_ and Co_3_O_4_ respectively. High resolution transmission electron microscope (HRTEM) image of Ni_1_Co_2_ sponge (which in the later section is discussed as the best material among the other sponges towards OER) is carried out and is shown in Fig. 2F. The HRTEM image has been taken after dispersing the sponge in acetone and drop casting it on a copper grid. The image shows that the sponge still keeps the structural integrity and porous nature. The elemental mapping performed using this Scanning TEM (STEM) - EDS is shown in Fig. 2G,H. Uniform distribution of Ni and Co throughout the sponge is evident from the mapping. It indicates the uniform alloying of the formed Ni_1_Co_2_ sponge.

In order to have a better insight about the chemical states of the sponges at the surface, XPS studies were conducted on as prepared Ni_1_Co_2_ sample without ion etching. The characteristic peaks of Ni, Co and O are observable in the survey spectrum. The XPS spectra of Co2p and Ni2p are shown in the Fig. 3. The broad Ni2p3/2 shows the presence of metallic Ni (~852.6 eV) and NiO (~853.7 eV). The multiple-split peaks in between 870–885 eV also indicate the presence of oxides (NiO) in the sample. The presence of multiple oxidation states is evident in the case of Co2p spectrum too (Fig. 3B). This indicates that a small percentage of surface oxidation has occurred during the sponge formation, though the amount is negligibly small to detect using wide angle XRD. The plasma treated surface etching followed by EDS studies conducted on Ni_1_Co_2_ sponges indicate that the presence of oxygen is limited to the surface of the sponge (~50 nm from the surface, supporting information S5). Further to evaluate the exact thickness of the oxide layer formed on the surface Rutherford back scattering (RBS) experiment is performed on the Ni_1_Co_2_. Figure 3C,D shows the RBS data taken at some of the energies. The oxygen peak starts appearing at 3.036 MeV and disappears after 3.078 MeV. All the spectra are simulated with three layered structure using SIMNRA software. From the RBS data we found out that the oxygen is only up to a depth of nearly ~44 nm, correlating with EDS studies.

### Electrochemical OER Studies

Linear sweep voltammograms (LSVs) for oxygen evolution reaction on various Ni and Co based samples are performed and the results are shown in Fig. 4A. The Ni_1_Co_2_ has shown highest activity (low onset potential and high current density) towards OER. The Electrocatalytic activity of Ni_1_Co_2_ before and after pre- oxidation is shown in Figure S6. After the pre-oxidation, OER activity of all the sponges increased drastically due to the formation of elecrtocatalytically active oxide rich surface, where the oxide layer will enhance the adsorption kinetics while inner metallic core will contribute to the conductivity of carriers. The Tafel slopes of different samples after pre-oxidation are given in Fig. 4C. Tafel slopes are calculated as 96.4 mV/dec, 94.8 mV/dec, 94.3 mV/dec, 92.8 mV/dec and 87.3 mV/dec for Ni, Co_3_O_4_, Ni_2_Co_1_, Ni_1_Co_1_ and Ni_1_Co_2_ respectively. The lower Tafel slope value of Ni_1_Co_2_ suggests its higher OER activity. During the OER measurements, Ni_1_Co_2_ shows a significantly low over potential (1.629 V) to reach a current density of 10 mAcm^−2^ in comparison to that of Ni_1_Co_1_ (1.654 V), Co_3_O_4_ (1.674 V), Ni_2_Co_1_ (1.686 V) and Ni (1.744 V) as shown in Fig. 4D. The stable Ni-Co-oxide/Ni-Co alloy hetero structure is responsible for the augmented electrocatalytic properties of Ni_1_Co_2_. Figure 4B shows the LSVs for the effect of scan rate on peak current for the Ni_1_Co_2_ sample. Inset of the Fig. 4B shows the plot of peak current vs scan rate. Thin layer diffusion occurring at the electrodes is proved by scan rate dependent peak current measurements (supporting information Figure S7). The slope of the log (peak current) vs log (scan rate) is found to be ~0.98, indicating the thin layer diffusion as reported in the literature^46^.

To calculate the charge transfer resistance during OER measurements electrochemical impedance spectroscopy (EIS) is conducted on various samples using a three electrode system by applying a potential of 1.594 V in 0.1 M KOH. The frequency of the ac voltage was swept in the range of 100 KHz to 1 Hz, and the impedance data were fitted to the semicircle for calculating the R_ct_ values. The R_ct_ values for different sponges are found to be 710 Ω, 514 Ω, 407 Ω, 287 Ω and 23 Ω for Ni, Co_3_O_4_, Ni_2_Co_1_, Ni_1_Co_1_ and Ni_1_Co_2_ respectively (Supporting information, Figure S8). The less charge transfer resistance of Ni_1_Co_2_ is in concordance with the enhancement in its OER performance. The OER performance of Ni_1_Co_2_ is compared with that of the benchmarked catalyst IrO_2_ and is shown in supporting information Figure S9. The higher activity of Ni_1_Co_2_ towards OER is evident from this graph. Further, the Faradaic efficiency of the Ni_1_Co_2_ electrode is calculated as 92% using Rotating Ring Disc Electrode (RRDE) voltammetry (the details are given in supporting information, Figure S10). The RRDE experiment confirms the formation of O_2_ at the anode, whilst to further confirm the formation of O_2_, a ‘blue bottle’ experiment is conducted where the oxidation of *leuco*- methylene blue to methylene blue is shown as a confirmatory test for the formation of O_2_ at the anode (please see the details in supporting information Figure S11 and Video S1).

### Electrochemical HER studies

The above discussed Ni_1_Co_2_ based sponge is studied for it’s HER catalytic performance in alkaline media. The alkaline HER catalytic activities of various sponges namely, Ni, nitrogen doped graphene powder (NG), Ni_1_Co_2_, and Ni-NG, are compared and it is shown in supporting information, Figure S12. It is observed that the HER catalytic activity of Ni sponge is improved with the incorporation of NG (a recent study indicates that nitrogen doping can introduce more HER active sites in graphene resulting in to a higher HER efficiency with higher amount of nitrogen. Hence NG can bring HER activity in Ni sponge and this is in tune with the report of Fei *et al*.^47^. Hence a water electrolysis full cell can be developed without the aid of benchmarked precious metal/metal oxide catalysts. The porous morphology of Ni-NG is shown in supporting information Figure S13. The XPS survey spectrum is shown in the supporting information indicating the presence of Ni, carbon (C), nitrogen (N) and oxygen. The presence of oxygen and multiple oxidation states of Ni in Ni2p spectrum indicate the traces of NiO in the Ni-NG sponge (supporting information Figure S14).

HER polarization plots measured in 0.1 M KOH with Ni-NG and benchmarked Pt/C are shown in Fig. 5A. The over potentials measured at 10 mAcm^−2^ for Ni-NG and Pt/C are found to be −0.246 V and −0.126 V respectively. This value obtained for Ni-NG is found to be far better in terms of stability and over potential than that of the previously reported Ni based catalysts, and comparable with that of the recently reported other HER catalysts^48^,^49^. The Tafel slopes calculated for Ni-NG and Pt/C are 44 mV/dec and 36 mV/dec, respectively. The lower Tafel value of Ni-NG suggests the favorable HER occurring at Ni- NG.

### Water electrolysis cell setup using Ni_1_Co_2_ and Ni-NG

**Figure 1:**
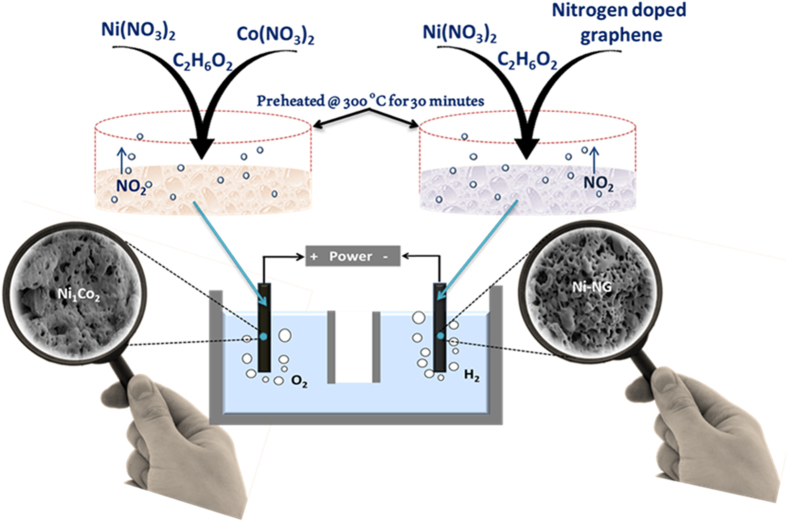
Scheme of the Ni-Co and Ni-NG catalysts based water electrolysis cell. The cell is working at ~1.59 V and the generation of 2:1 ratio H_2_ and O_2_ is sketched in the figure. The chemistry of sponge synthesis is also depicted.

**Figure 2:**
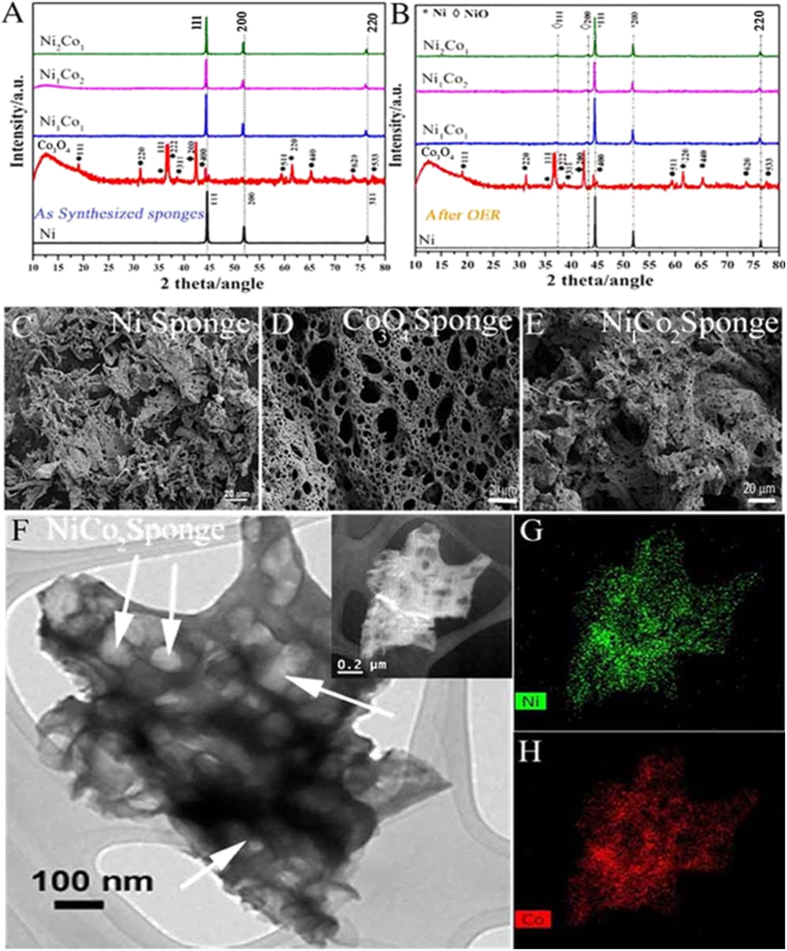
(**A**,**B**) XRD patterns of different types of Ni and Co sponges before and after OER experiments. (**C**–**E**) FESEM images of Ni, Co_3_O_4_ and Ni_1_Co_2_ sponges respectively. (**F**) HRTEM image of Ni_1_Co_2_ (arrows represent the pores in the sponge), (**G**,**H**) are the STEM-EDS elemental mapping for Ni and Co in the corresponding TEM image shown in the inset of (**F**) respectively.

**Figure 3:**
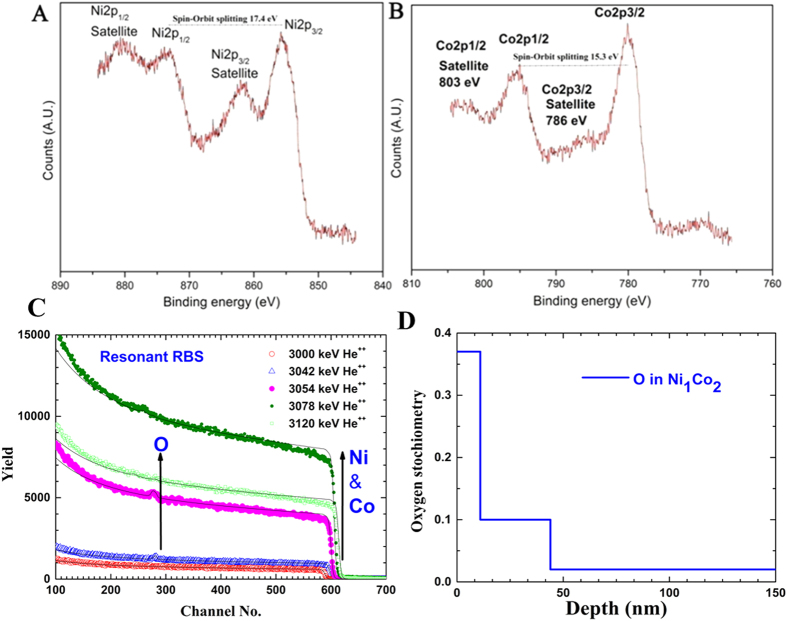
(**A**,**B**) Ni 2p and Co 2p XPS spectra of Ni_1_Co_2_ sponge. Both the spectra show the presence of oxides in the samples. (**C,D**) RBS spectra of Ni_1_Co_2_ showing the oxygen depth profile in the sponge.

**Figure 4:**
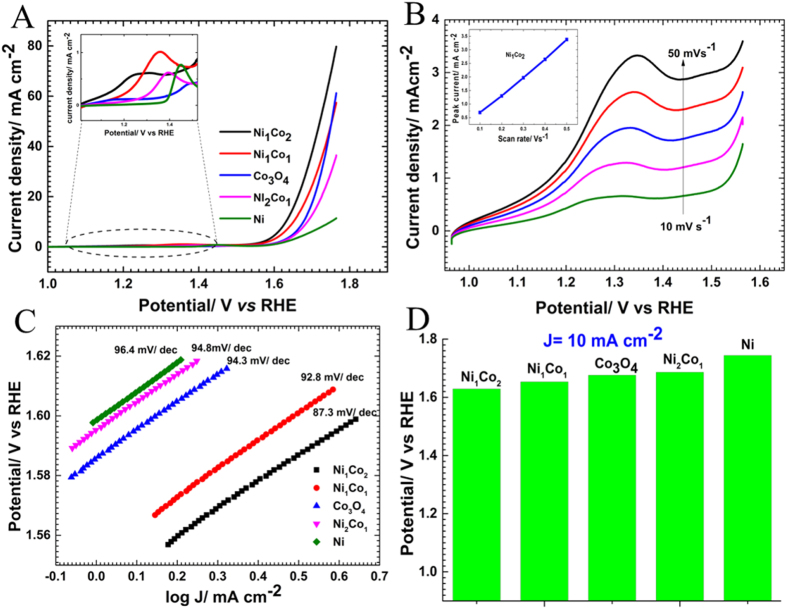
(**A**) LSVs for the comparison of oxygen evolution activities of different catalysts in 0.1 M KOH, scan rate 10 mV/s (inset magnifies the area marked by dotted circle). (**B**) LSVs of Ni_1_Co_2_ at various scan rate ranging from 10 mv/s to 50 mV/s, inset shows the plot of peak current vs scan rate showing the linearity. (**C**) Tafel plot derived from (**A**) for various catalysts, and (**D**) potential needed to achieve the current density 10 mAcm^−2^ (over potentials of various electrodes).

**Figure 5:**
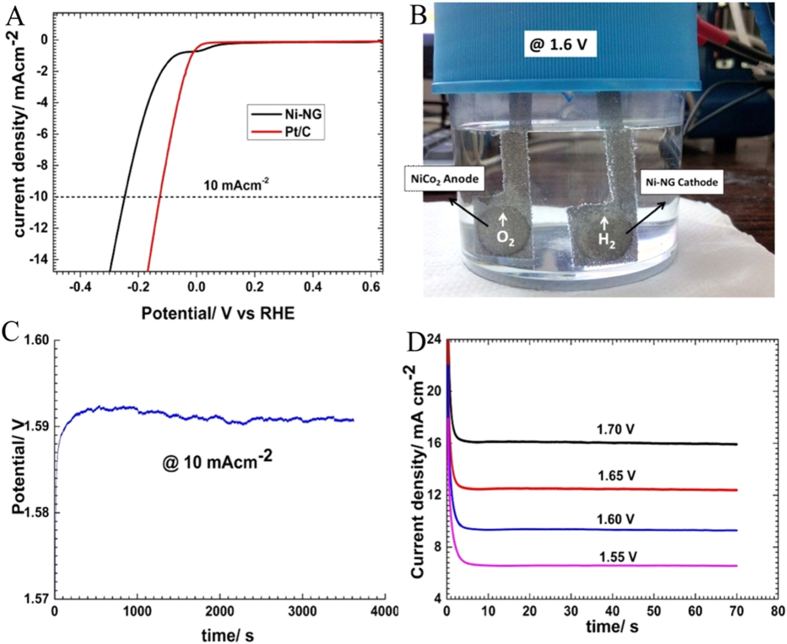
Hydrogen evolution activity Ni-NG catalyst in comparison with Pt/C, (**B**) Image shows the production of O_2_ and H_2_ at an operating potential of 1.59 V using the freestanding spongy catalysts electrodes developed and inserted in to a commercial Ni sponge using hydraulic pressurizer, (**C**) Chronopotetentiometric response at 10 mAcm^−2^ showing the stability in performance, and (**D**) Chronoamperometric analysis at different operating voltages.

Recently Dai *et al*. reported a water electrolysis cell (1.5 V) operated with their catalyst as cathode and Ni- Fe layered double hydroxide as anode^50^. Here, we demonstrate the water electrolysis cell with our own catalysts working at similar potentials. A two electrode cell is developed as shown in Fig. 5B by incorporating the anode material (Ni_1_Co_2_ sponge) and cathode material (Ni-NG sponge) on a commercial Ni sponge (loading of 10 mgcm^−2^) by applying a pressure of 1 MPa using a hydraulic pressurizer (schematic of the same is shown in Fig. 1). The photographic image of the sponge and the electrode morphology are shown in supporting information Figure S15. The advantage of such a fluffy and spongy catalyst is the possibility of its direct incorporation into other electrodes without the aid of any external binder (use of binder will increase the resistance). The electrolysis cell shows a very high stability >10 hr at a current density of 10 mA cm^−2^ as shown in Fig. 5C. In order to check the current density at different potentials chronoamperometric measurements are performed and are shown in Fig. 5D. The stability of the electrodes in different working potentials is evident from these studies which ensure the commercial feasibility of this water electrolysis set up.

## Conclusions

A bulk method for the development of free standing metallic sponges with the possibility of one step controllable alloying is discussed. Out of various stoichiometric combinations of Ni-Co based sponges tested, Ni_1_Co_2_ sponge showed enhanced OER activity (low over potential 1.629 V at 10 mAcm^−2^), long stability, low tafel slope (87.3 mV/dec), and high Faradaic efficiency, and its OER performance is comparable to precious metal oxide based benchmarked catalyst. It is further proven that Ni_1_Co_2_/oxide heterostructure is playing the key role in augmented OER process. The synthesis strategy is extended to develop graphene based HER catalyst, where the developed Ni-NG sponge’s HER performance is comparable to that of Pt/C- a well-known commercially available HER catalyst. A complete water electrolysis cell is designed with the developed catalysts and its performance is studied. The enormous stability (>10 hours) and performance (10 mAcm^−2^ at 1.59 V) of the developed full cell indicates the feasibility of these freestanding electrodes based cells for commercial energy production applications.

## Methods

### Synthesis of Different Types of Sponges

36 g of Ni(NO_3_)_2_.6H_2_O (Sigma-Aldrich) was added to 18 mL of EG (C_2_H_6_O_2_) (Sigma-Aldrich) and ultra-sonicated for 1 hour. After that the resultant mixture was poured into a cylindrical crystalline glass dish, which was pre-heated to a temperature of 300 °C for 30 minutes by using a hot plate in the ambient atmosphere. A sudden introduction of reactants in to the hot glass dish leads to the evolution of NO_2_ gas bubbles and subsequent production of fluffy Ni sponge. Cobalt oxide (Co_3_O_4_) sponge was prepared by the reaction of Co(NO_3_)_2_.6H_2_O (36 g) and EG (18 mL) by using the same procedure mentioned above for Ni sponge. The same synthesis route is followed for the preparation of Ni-Co alloy sponges. Here reactants Ni(NO_3_)_2_.6H_2_O and Co(NO_3_)_2_.6H_2_O (sigma-Aldrich), were mixed together taking a total amount of 36 g in the weight ratio of 1:1, 1:2 and 2:1 and the resulting sponges named as Ni_1_Co_1_, Ni_1_Co_2_ and Ni_2_Co_1_. For the synthesis of Ni-NG, 36 g of Ni(NO_3_)_2_.6H_2_O (Sigma-Aldrich) and 100 mg of NG(prepared by heating GO with melamine (1:5 ratio) for 3 hrs at 800 °C)^51^ were added to 18 mL of EG (C_2_H_6_O_2_) (Sigma-Aldrich) and ultra-sonicated for 1 hour. The resultant mixture was poured in to a cylindrical dish preheated for 1 hour leads to the formation of Ni-NG sponges.

### Material Characterization

XRD of the powder samples were taken using Bruker X-ray powder diffractometer with Cu-Kα radiation. XPS analysis was done by using PHI 5000 Versa Probe ULVAC instrument and CARLZEIXX- SUPRA 55 VP was used for FESEM analysis. HRTEM images and selected area diffraction patterns (SAED) were performed using JEM 2100 field emission gun transmission electron microscope with an accelerating voltage of 200 kV. Rutherford backscattering spectra are taken by using MeV Helium ions from 1.7 MV tandetron accelerator at IGCAR, Kalpakkam, India.

### Electrochemical Experiments

All the electrochemical experiments were conducted in 0.1 M KOH with a glassy carbon electrode as the working electrode, platinum auxiliary electrode, and Ag/AgCl as reference electrode in a conventional three- electrode cell. The measured potentials vs Ag/AgCl were converted to the reversible hydrogen electrode (RHE) scale according to the Nernst equation,


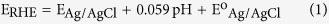


where, E_RHE_ is the converted potential vs. RHE, E^o^_Ag/AgCl_ = 0.197 at 25 °C, and E_Ag/AgCl_ is the experimentally measured potential against Ag/AgCl reference.

Catalyst inks were prepared by sonication of a mixture of 4 mg catalyst powder (sponge), 50 μL nafion (5 wt% dispersion in lower aliphatic alcohol and water) and 1 mL isopropanol/water mixture. The ink was drop casted onto the readily polished glassy carbon (GC) electrode to obtain a loading of 0.28 mg cm^−2^. Prior to the test, all Ni-containing catalysts (sponges) were electrochemically activated (chrono-amperometry for 2 hours at 1.564 V) to enhance the electrocatalytic activity of sponges. The pre-treatment of the electrodes will help to form a stable oxide layer which in turn results in to a saturated OER current.

## Additional Information

**How to cite this article**: Vineesh, T. V. *et al*. Controllably Alloyed, Low Density, Free-standing Ni-Co and Ni-Graphene Sponges for Electrocatalytic Water Splitting. *Sci. Rep.* **6**, 31202; doi: 10.1038/srep31202 (2016).

## Supplementary Material

Supplementary Video S1

Supplementary Information
