# Supplementary material for: Controllably Alloyed, Low Density, Free-standing Ni-Co and Ni-Graphene Sponges for Electrocatalytic Water Splitting
Source: Sci Rep. 2016 Aug 11;6:31202. doi: 10.1038/srep31202 (PMC4980622; doi:10.1038/srep31202)
Supplement: Supplementary Information [file srep31202-s2.pdf]

Supporting Information

Controllably Alloyed, Low Density, Free-standing Ni-Co and Ni-Graphene Sponges for Electrocatalytic Water Splitting

Thazhe Veettil Vineesh, Suhail Mubarak, Myung Gwan Hahm, V. Prabu, S. Alwarappan,* & Tharangattu N. Narayanan*

T. V. Vineesh, V.Prabu, Dr. S. Alwarappan,
CSIR-Central Electrochemical Research Institute (CSIR-CECRI),
Karaikudi – 630 006, India.
salwarap@gmail.com

Dr.Myung G. Hahm
School of material science and Engineering,Inha University,
100 Inharo, Nam- gu, Incheon, 22212, Korea.

T. V. Vineesh, M. Suhail, Dr. T. N. Narayanan,
TIFR-Centre for Interdisciplinary Sciences (TCIS)
Tata Institute of Fundamental Research
Hyderabad – 500 075, India.
(*Corresponding authors: tn_narayanan@yahoo.com or tnn@tifrh.res.in or salwarap@gmail.com)

Supporting Information Video S1 (attached)

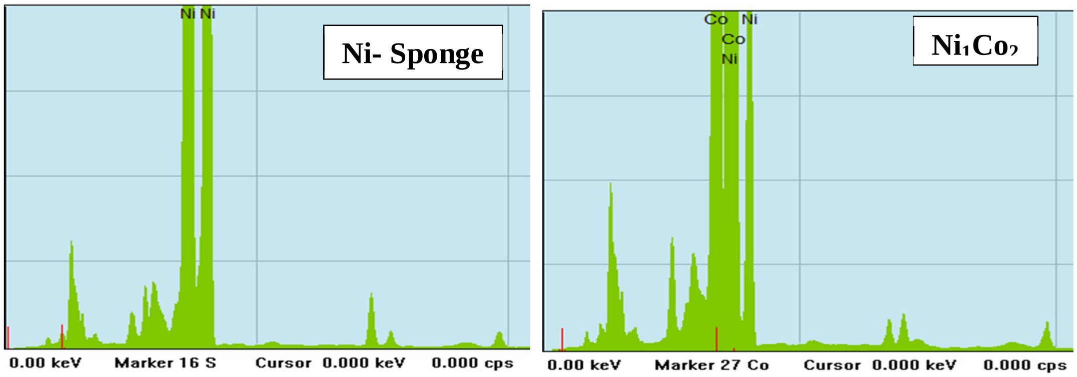

Sponge	Ni Weight %	Co Weight %
**Ni alone**	**100**	**0**
**Ni_1Co_1**	**49.30**	**50.70**
**Ni_2Co_1**	**66.36**	**33.64**
**Ni_1Co_2**	**32.99**	**67.01**

Figure S1. XRF spectrum ofNi and Ni_1Co_2alloy. The column shows the percentage of Ni and Co in the different types of alloy sponges.

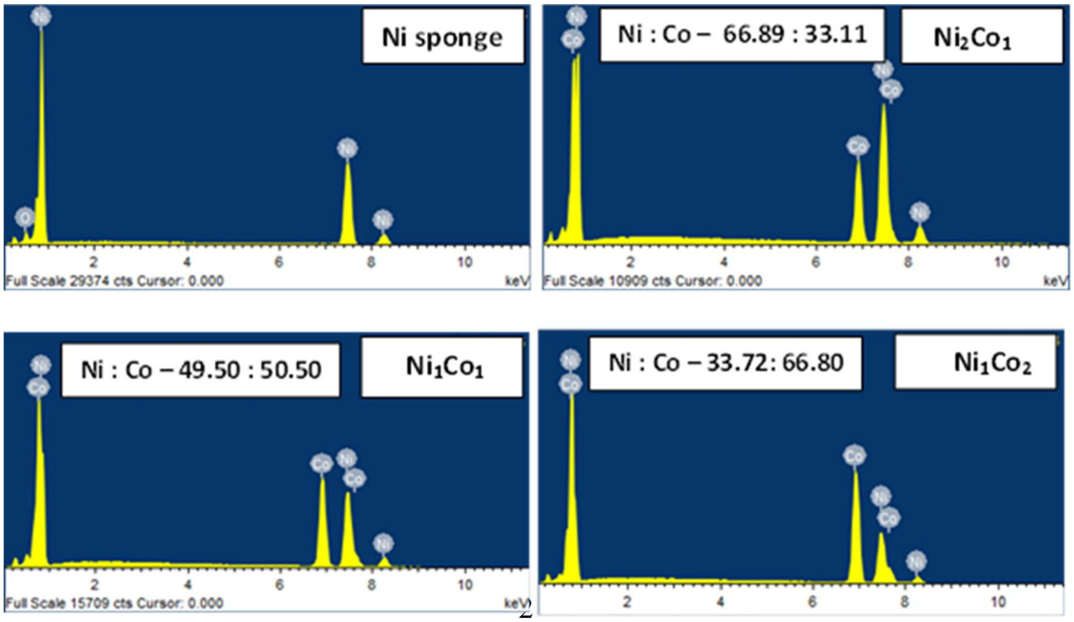

Figure S2.FESEM- EDS spectra of different spongessynthesized by using nickel and cobalt.

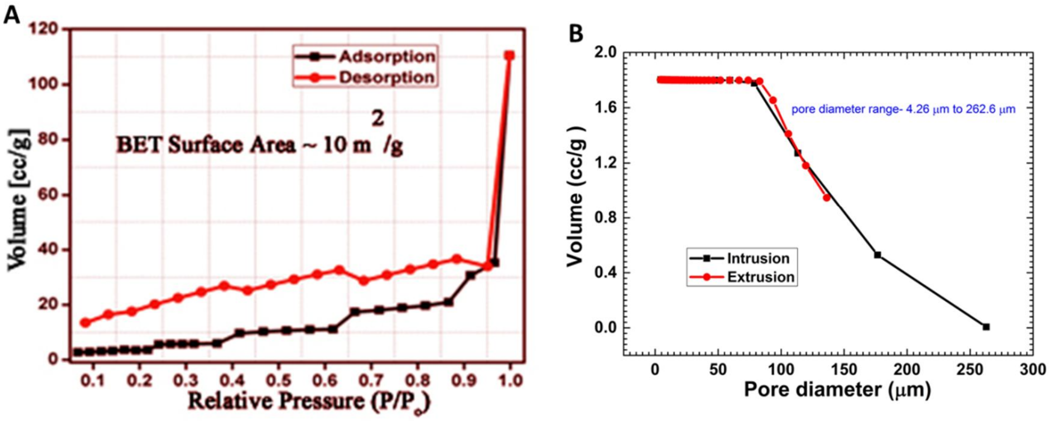

Figure S3. (A) BET surface area analysis and (B) mercury porosimetry experiment on Ni_1Co_2 sample.

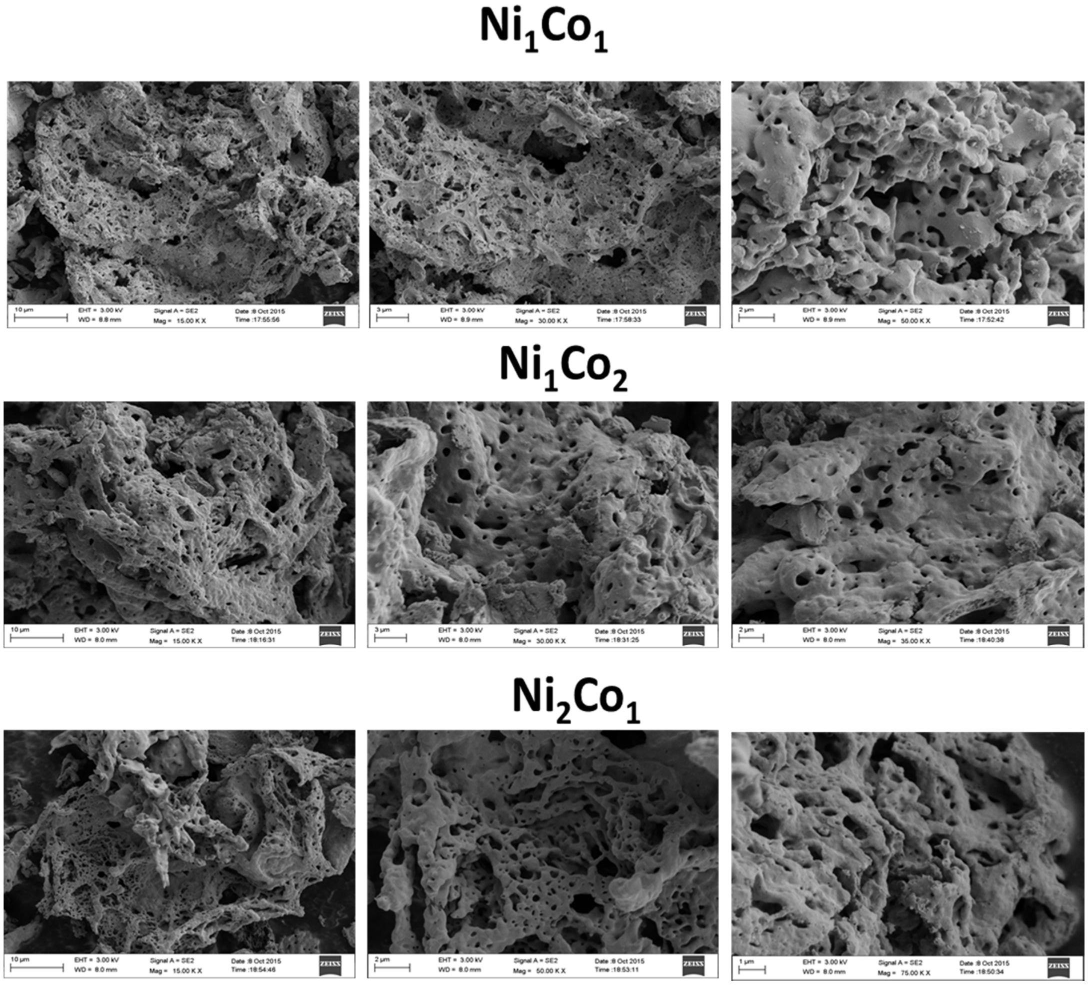

Figure S4. FESEM images different types of alloys at various magnifications.

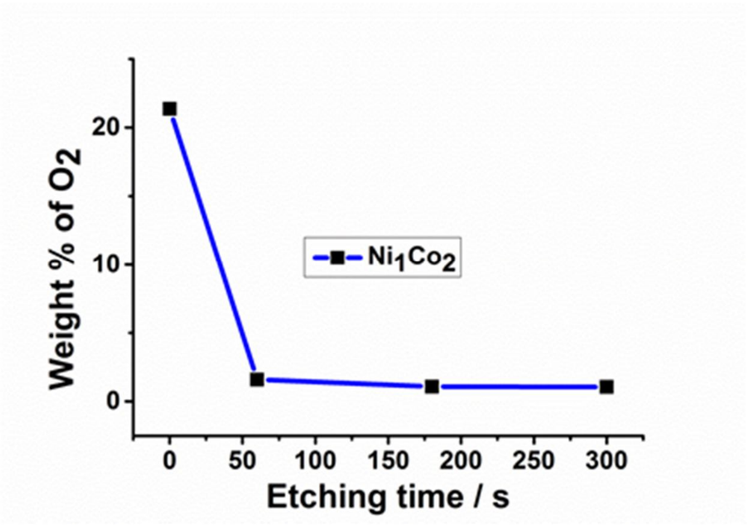

Figure S5.Oxygen amount mapped using EDS for various plasma etching intervals. The plasma treatment on Ni_1Co_2 sponge is conducted using PC- 2000 plasma etcher (South Bay Technology) for various etching times, and then recorded the oxygen amount using

EDS. It is to be noted from the figure that the oxygen content is drastically decreased from 21.37% (0 s etching) to 1.07% (300 s etching), after plasma treatment. This indicates that the prominent oxygen content is limited to the surface of the sponge.

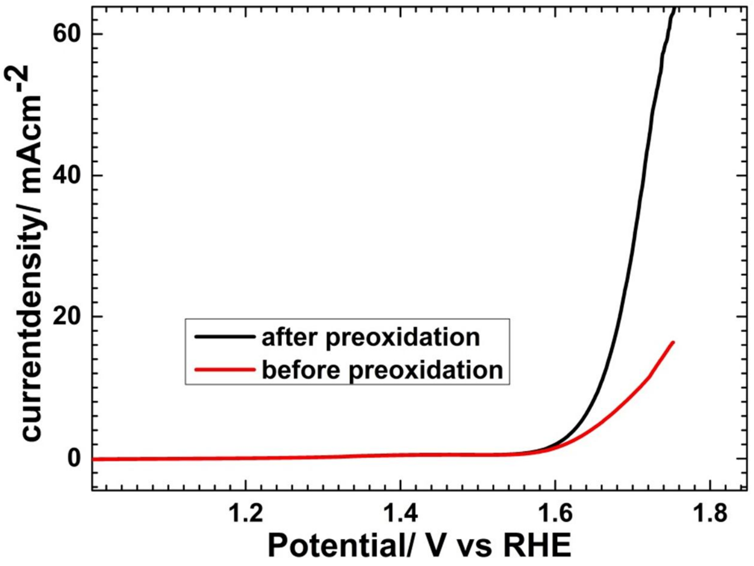

Figure S6. LSVs for Ni_1Co_2 sponge before and after pre-oxidation (the pre-oxidation was carried out at 1.664 V for 2hrs).

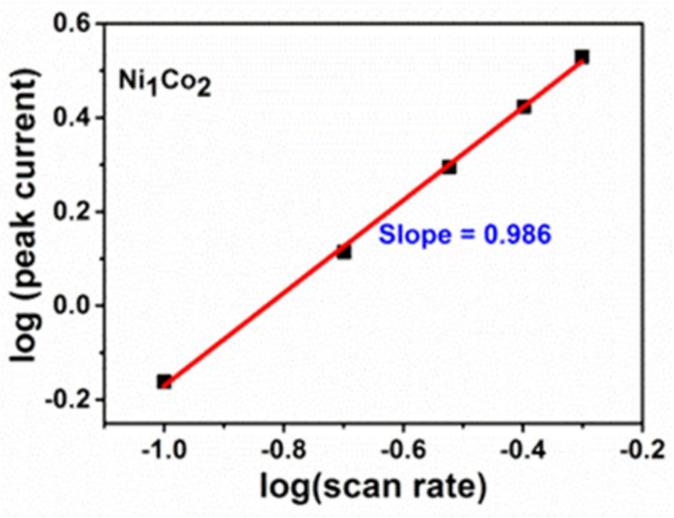

Figure S7.The plot for confirming the thin layer diffusion in the sample

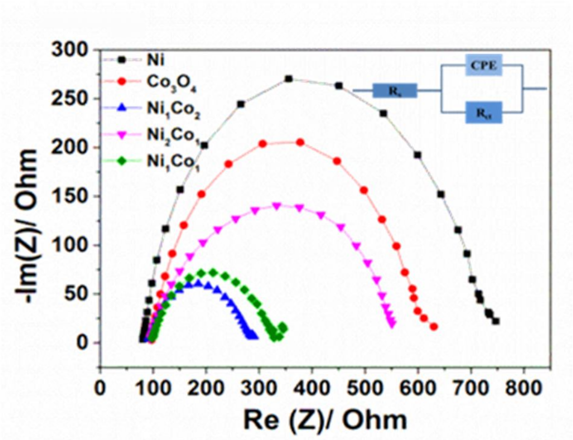

Figure S8. The electrochemical impedance spectra of various types of sponges (inset shows the corresponding Randles circuit, where R_s corresponds to the solutions resistance, CPE constant phase element (related to the interfacial capacitance which is in parallel to OER charge transfer resistance R_{ct}), and R_{ct} is the charge transfer resistance).

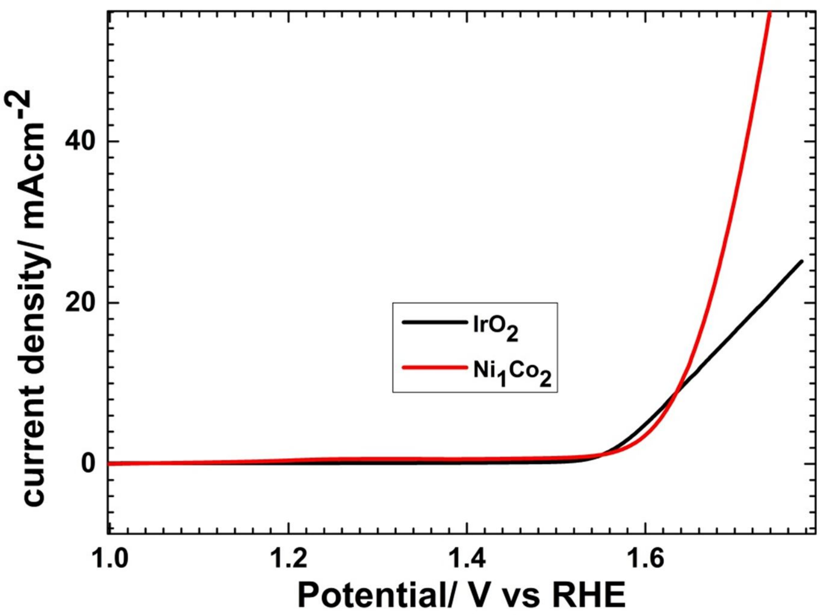

Figure S9.Comparison of electrocatalytic OER activity of Ni_1Co_2 with benchmarked IrO_2 catalyst.

Calculation of Faradaic efficiency from RRDE voltammetry

In order to calculate Faradaic efficiency, the collection efficiency of the ring electrode (Pt) needs to be determined. RRDE experiments were carried out using a glassy carbon disk-Pt ring RRDE electrode connected with BioLogic work station.

RRDE- Collection efficiency calculation:

In a typical Rotating Ring Disc Electrode (RRDE) collection efficiency experiment, the complete product generated at the disk electrode will not reach the ring electrode. The percentage of material which is collected at the ring electrode is often called the "collection efficiency" of the RRDE. One may empirically measure the collection efficiency of a specific RRDE before using it for any quantitative work. This is normally carried out using a well-defined electrochemical system such as the ferricyanide- ferrocyanide redox couple. This system can be used to measure stable collection efficiency at rates between 400 and 2000 rpm. The ring collection efficiency was calculated to be 37% (or expressed as 0.37), as measured using a $[Fe(CN)_6]^{3-/4-}$ redox couple. The disc electrode is swept from 0.6V to -0.2V *vs.* Ag/AgCl and the ring potential is kept at 0.23V *vs.* Ag/AgCl. The collection efficiency (N_{CL}), calculated from the current response of $K_3Fe(CN)_6$ (5 mM) in 0.1M KCl was found to be 0.37 ± 1%. Ar gas was purged into the electrochemical cell for 30 min before the experiment and remained the Ar flow throughout the experiment.

Faradaic efficiency calculation:

In order to calculate the faradaic efficiency, 10 μL of the ink solution containing 4mg/mL Ni_1Co_2 catalyst was drop-cast on the glassy carbon (GC) disk electrode. When the Ni_1Co_2 loaded GC disk electrode (working electrode) potential was swept in anodic potentials, oxygen is generated from the working electrode and the produced oxygen will moved towards the platinum ring electrodes due to the rotating action (laminar flow) of the electrode

(1600rpm). The platinum ring electrode is held at -0.5V vs. Ag/AgCl, which will facilitates the reduction of oxygen[2]. The Faradaic efficiency can be calculated based on the ratio of disk and ring currents using the following equation:

$$\varepsilon = \frac{2i_r}{i_d N_{CL}}$$

where, i_r = ring current (A); i_d = disk current (A); N_{CL} = collection efficiency (%), ε = Faradaic efficiency (%).

The faradaic efficiency calculated from the RRDE study (figure given below) is found to be 92%.

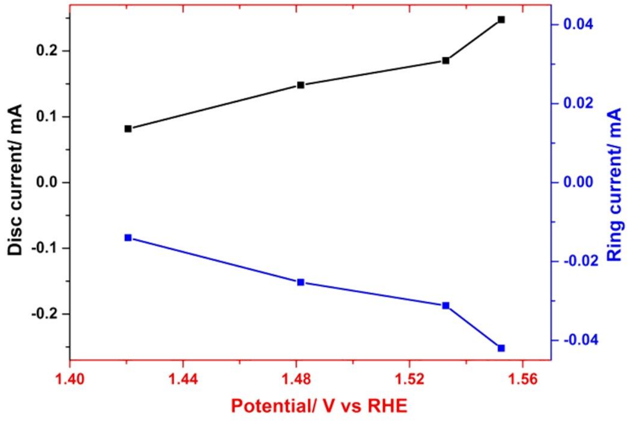

Figure S10. Determination of Faradaic efficiency of Ni_1Co_2 catalyst.

'Blue Bottle' Experiment:

To further analyse the gas produced in anode (OER reaction), an alkaline solution [1M solution (pH ~14)] of KOH containing glucose (Sigma Aldrich] is taken. Here glucose will act as reducing agent to change the colour of added methylene blue (sigma Aldrich) dye from blue to colourless *leuco*- methylene blue. Shaking/ introduction (purging) of external oxygen to this solution containing *leuco*- methylene blue will lead to the oxidation back to methylene blue - leading to regaining of its parent blue colour. When the dissolved oxygen has been fully consumed, methylene blue will slowly reduce back to its colourless form by the remaining

glucose. In the present experiment, the above colourless solution is saturated with N_2 gas and showed that even shaking the solution does not give any colour change. Further, oxygen gas produced in a anode (Ni_1Co_2 anode) of MEA of Fuel Cell set up (5 cm^2 Single Cell fixture with monopolar plates in GRAPHITE and O-Ring) is purged in to the colourless solution of glucose containing methlyne blue and noticed a colour change in the solution indicating the presence of oxygen gas (video of the whole process is attached where the 'light blue' colouration near to the purging tube is due to the oxidation of reduced methlyne blue and it confirms the formation of oxygen in anode).

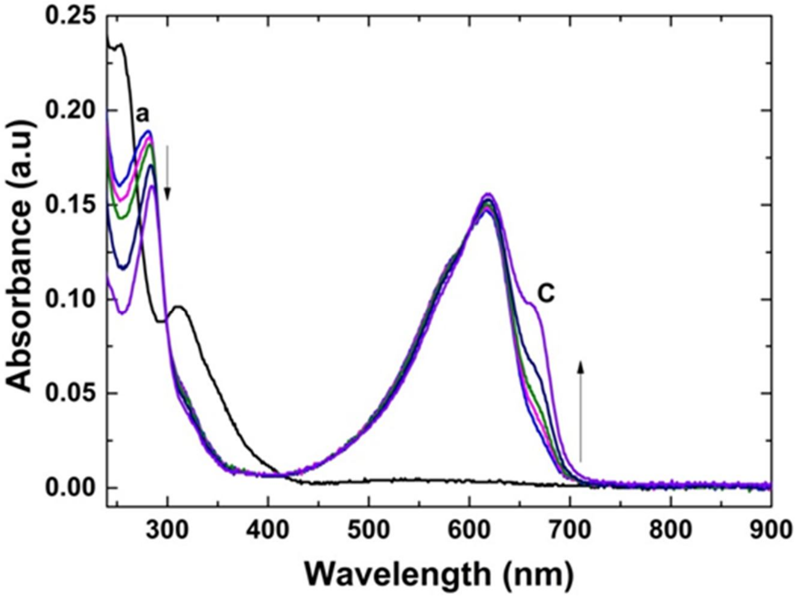

Figure S11. UV– Vis spectra of leuco- MB in presence of oxygen (the peak marked as ‘a’ corresponds to leuco- methylene blue and ‘c’ corresponds to methylene blue).

Further, UV- Vis spectroscopy is used to confirm the transformation of *leuco*- methylene blue to methylene blue due to the presence of oxygen. The UV Vis spectra of *leuco*- methylene blue solution after various times (black to violet) of oxygen purging are given in the figure S11. In the presence of oxygen (the gas coming out from our MEA setup) there is decrease in intensity of peak corresponds to *leuco*- methylene blue (peak a) centred at

282 nm), and an increase in peak (peak marked C) intensity corresponds to methylene blue (664 nm). This clearly confirms the production of oxygen gas from the water electrolyser set up.

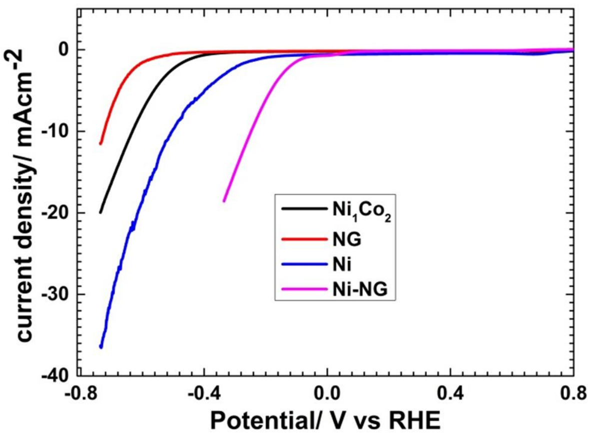

Figure S12. HER activity of different types of materials used in this study.

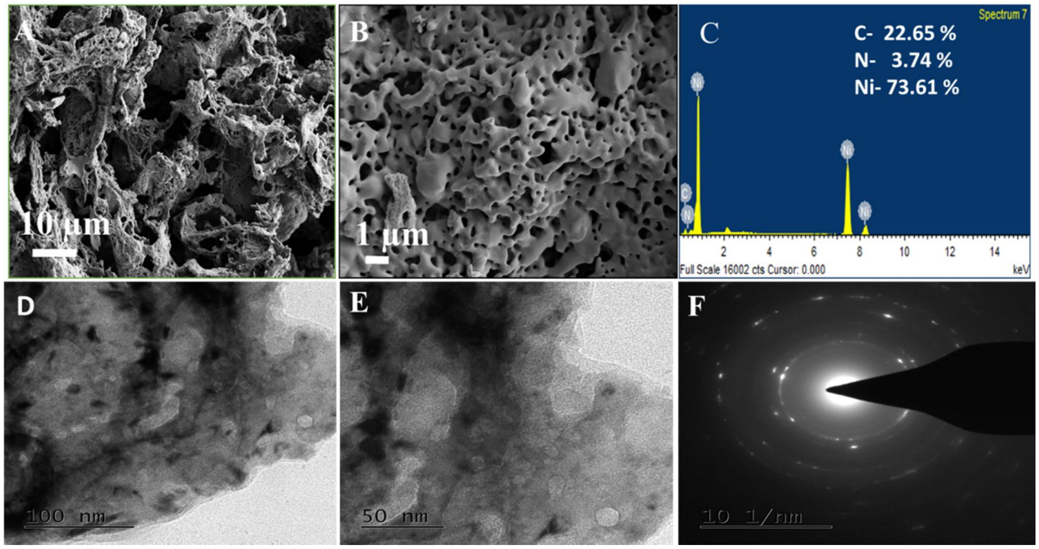

Figure S13. FE-SEM images of Ni- NG (A & B) and corresponding EDAX spectrum (C). HRTEM images of Ni-NG at different magnification (D & E) and corresponding SAED pattern (F).

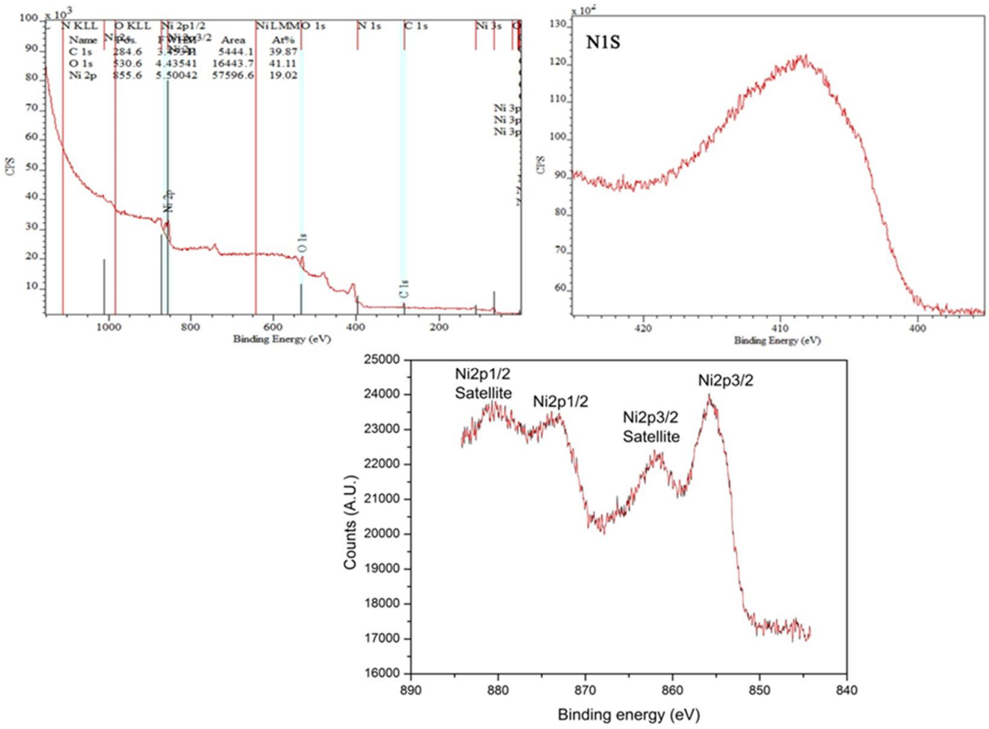

Figure S14. (A) XPS survey spectra of Ni NG sponge. Survey scan showing the presence of Ni, C, O and N. (B) N1s and (C) Ni2p spectrum showing the presence of multiple oxidation states of Ni.

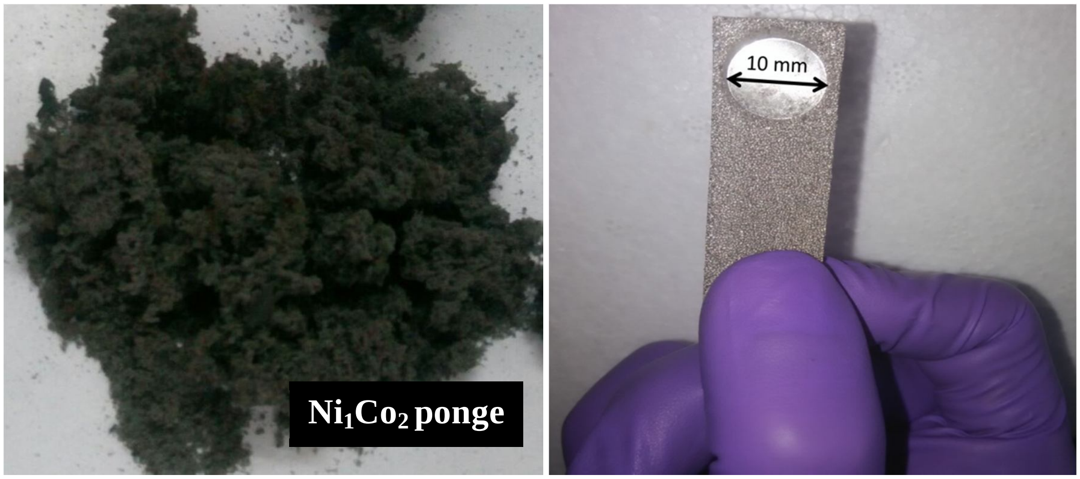

Figure S15. (A) Photographic image of Ni_1Co_2 sponge and (B) after pressing the sponge on nickel foam by applying a pressure of 1 Mpa.

References:

1. Wang, X. & Otto, S. W. Optical methods for sensing and imaging oxygen: materials, spectroscopies and applications. *Chem. Soc. Rev.* **43**, 3666–761 (2014).
2. Chen, Z., Coleman X. K. & Bruce E. K. Facet-dependent activity and stability of Co_3O_4 nanocrystals towards the oxygen evolution reaction. *Phys. Chem. Chem. Phys.*, **17,** 29387—29393 (2015).
